# Pitfalls and Tips for Statistical Methods in Epidemiology: A New Series of Special Articles Has Started

**DOI:** 10.2188/jea.JE20190360

**Published:** 2020-04-05

**Authors:** Takeo Fujiwara

**Affiliations:** Department of Global Health Promotion, Tokyo Medical and Dental University, Tokyo, Japan

The number of papers related to epidemiology and researchers who engage in epidemiology is substantially increasing. PubMed search using the keyword “epidemiology” demonstrated that 36,054 and 149,395 papers were published in 2000 and 2018, respectively (ie, an increase of more than four times in the last two decades). For epidemiology studies related to Japan, the number of available papers observed using PubMed search for “epidemiology” and “Japan” increased from 1,079 in 1998 to 5,499 in 2018 (ie, a five-fold increase). Additionally, the members of the Japan Epidemiology Association increased from 1,115 in 1998 to 2,305 in 2018 (ie, more than doubled in two decades). Moreover, the reason for this increase may be owing to the easier access to cost-free open databases and faster permission processes, such as those for accessing electronic medical records or administrative records, as well as the rapid advancement of statistical methods, which are relatively easy to run using statistical software, such as SAS, Stata, SPSS, or R. Moreover, recent developments in the utility of “big data” and “artificial intelligence” have accelerated this trend.

Consequently, this trend must be welcomed. In addition, following the experience of working with new researchers of epidemiology, numerous tips and pitfalls have been identified that need to be summarized and noted before applying new statistical methods in epidemiology. For example, directed acyclic graphs (DAGs), multiple imputations, or propensity score matching analysis are extensively utilized, but there are several considerations. Furthermore, numerous tips are useful but not exactly given in textbooks or papers. Therefore, for those who engage in epidemiology studies, a summary of these pitfalls and tips for these new methods would be beneficial. In this series of new special articles, it is planned to discuss the following epidemiologic methods for both analysis and measurement:

1) Causal diagrams (ie, DAGs) (in this issue)
2) Marginal structural model
3) Propensity score methods (matching and inverse probability weighting)
4) Mediation analysis
5) Machine learning

For the first special article in this theme, Suzuki et al summarized pitfalls and tips for causal diagrams, which are known as DAGs.^1^ DAGs were first introduced by Greenland, Pearl, and Robins in 1999,^2^ and they have been widely used by epidemiologist to select covariates. DAGs are a useful tool for summarizing the association between various variables, and numerous educational review articles have already been published.^3^ DAG was described in A Dictionary of Epidemiology, 5^th^ edition as follows: “A graphical display of causal relations among variables, in which each variable is assigned a fixed location on the graph (called a node) and in which each direct causal effect of one variable on another is represented by an arrow with its tail at the cause and its head at the effect. Direct noncausal associations are usually represented by lines without arrowheads. Graphs with only directed arrows (in which all direct associations are causal) are called directed graphs.”^4^ Therefore, we usually draw an arrow from explanatory variable “X” to outcome variable “Y,” but these main variables, as well as other covariates, need to be included in the graphical explanation.

Hence, the description continues as follows: “Graphs in which no variable can affect itself (no feedback loop) are called acyclic.”^4^ This description is very important, since we would like to infer the causal association. Thus, bidirectional association or cyclic association should not be used for a causal association. Further description is as follows: “Algorithms have been developed to determine from causal diagrams which sets of variables are sufficient to control confounding and for when control of variables leads to bias.”^4^ Use of DAGs allows researchers to carefully consider covariates, which include confounders, colliders, and mediators. Furthermore, these methods are useful not only for researchers of epidemiology, but also for the audience of the research.

In the current issue, Suzuki et al raises 10 pitfalls and tips for utilizing DAGs, which are useful and have been underreported the field of epidemiology. By utilizing this review article, researchers should feel more confident and avoid pitfalls when applying DAG to investigate research questions by properly selecting confounders and mediators. Furthermore, to advance the skills for employing new statistical methods in epidemiology, it is the anticipation of all *Journal of Epidemiology* Editors that this series will provide a meaningful learning opportunity for researchers.

## References

[1] Suzuki E, Shinozaki T, Yamamoto E Causal diagrams: pitfass and tips. J Epidemiol. 2020;30:153–162. 10.2188/jea.JE20190192 32009103 PMC7064555

[2] Greenland S, Pearl J, Robins JM Causal diagrams for epidemiologic research. Epidemiology. 1999;10(1):37–48. 10.1097/00001648-199901000-00008 9888278

[3] Fleischer NL, Diez Roux AV Using directed acyclic graphs to guide analyses of neighbourhood health effects: an introduction. J Epidemiol Community Health. 2008;62(9):842–846. 10.1136/jech.2007.067371 18701738

[4] Porta MS. *A Dictionary of Epidemiology*. 5th ed. New York, NY: Oxford University Press; 2008.

